# Protein carbonylation detection methods: A comparison

**DOI:** 10.1016/j.dib.2018.06.088

**Published:** 2018-07-03

**Authors:** Esra'a Alomari, Stefano Bruno, Luca Ronda, Gianluca Paredi, Stefano Bettati, Andrea Mozzarelli

**Affiliations:** a Department of Food and Drug, University of Parma, Parma, Italy; b Department of Medicine and Surgery, University of Parma, Parma, Italy; c Biopharmanet-TEC, University of Parma, Parma, Italy; d Istituto Nazionale Biostrutture e Biosistemi, Rome, Italy; e Istituto di Biofisica, Consiglio Nazionale delle Ricerche, Pisa, Italy

## Abstract

The data reported here are a comparison among four different methods for the detection of carbonylated proteins, a validated biomarker of oxidative stress. The reference samples were heart and kidney extracts of Guinea pigs transfused with hemoglobin-based oxygen carriers (Alomari et al. FRBM, [11]). We measured the carbonyl content of organ extracts by using i) the Levine spectrophotometric method, which takes advantage of the chromogenic reaction of carbonyl groups with 2,4-dinitrophenylhydrazine (DNPH), ii) a commercially available ELISA assay based on an anti-DNPH antibodies, iii) a commercially available Western blot method based on anti-DNPH antibodies and iv) an in-gel detection approach with the fluorophoric reagent fluorescein-5-thiosemicarbazide. The former two methods measure total protein carbonylation of a sample, whereas the latter two require an electrophoretic separation and therefore potentially allow for the identification of specific carbonylated proteins.

## Specifications Table

**Table:** 

Subject area	Biochemistry
More specific subject area	Oxidative stress
Type of data	Plots, figures
How data was acquired	SDS-PAGE, Western-blotting and gel imaging (Bio-Rad Laboratories, Inc., Hercules, California, U.S.A.), immunofluorescence, data graphical and statistical analysis (SigmaPlot, Systat Software, San Jose, CA, USA), plate reader (Halo LED 96 – Dynamica Scientific Ltd., Newton Pagnell, UK), absorption spectrophotometry (Cary4000, Varian/Agilent Technologies, Santa Clara, CA, USA)
Data format	Raw and analyzed
Experimental factors	Samples were stored at − 80 °C
Experimental features	ELISA assays, spectrophotometric measurements upon protein derivatization, SDS-PAGE, Western blot
Data source location	Parma, Italy
Data accessibility	Data is within this article

## Value of the data

• Protein carbonylation is a marker of oxidative stress and several methods have been devised and commercialized for its detection.
• Spectrophotometric methods yield results similar to the significantly more expensive immunochemical method.
• Hemoglobin- and myoglobin-containing samples can also be analyzed with spectrophotometric methods upon removal of the heme.
• Detection of carbonylated proteins upon electrophoretic separation can be carried out both by Western blot and by in-gel fluorophoric tagging – a significantly less expensive approach – with similar results.

## 1 Data

**Fig. 1:**
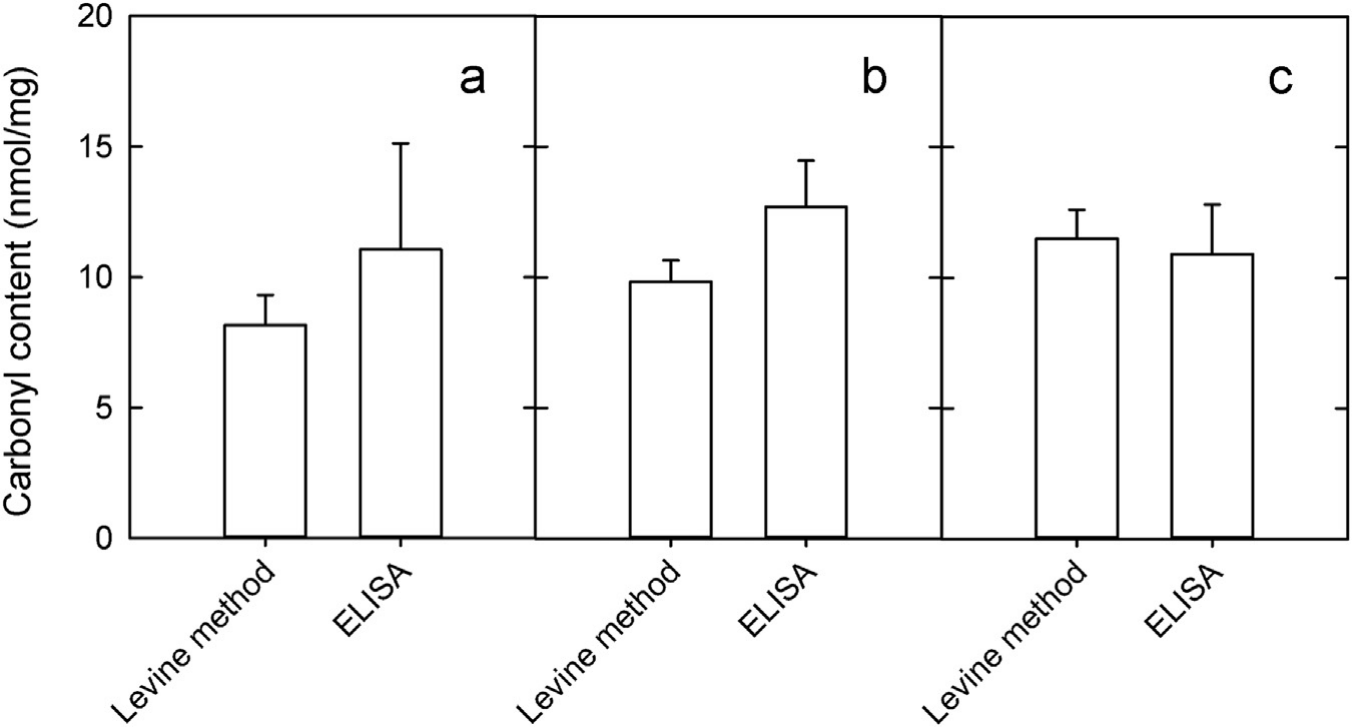
Determination of carbonyl content in animals that underwent autotransfusion (*n* = 5) **(a)**, animals treated with PEG-Hb^oxy^ (*n* = 3) **(b)** and animals treated with PEG-Hb^deoxy^ (*n* = 6) **(c)**. The error bars are the standard error of the mean.

The total carbonyl content as detected in heart tissue with the Levine method and the ELISA method (OxiSelect™ Protein Carbonyl ELISA Kit, Cell Biolabs, San Diego, CA, USA) is reported in Fig. 1 for the three animal groups described in FRBM [11]. No significant difference was observed between the two methods, with the ELISA method being more expensive.

**Fig. 2:**
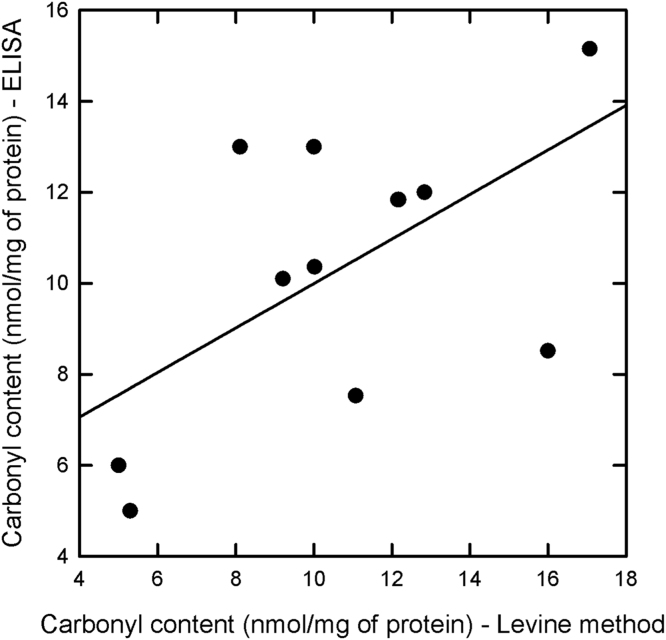
Scatter plot showing the correlation between the carbonyl content assessed for heart samples of Guinea pigs measured with the Levine method and with the OxiSelect^TM^ Protein Carbonyl ELISA Kit (Cell Biolabs, San Diego, CA, USA).

Values measured with two methods on each sample are correlated (Pearson correlation coefficient = 0.588, *p* = 0.0442) (Fig. 2).

**Fig. 3:**
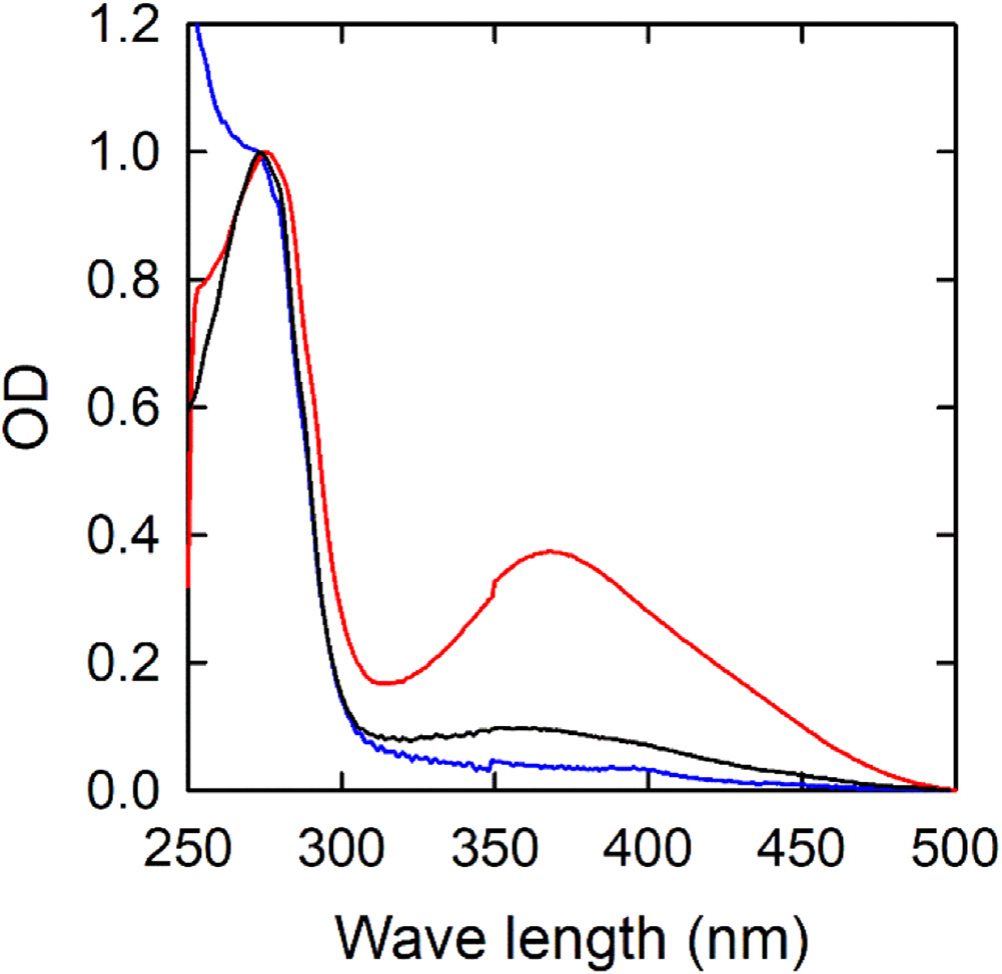
Spectra of a tissue extract of Guinea pig kidney treated with the protocol for the Levine method to detect carbonyl content. The red line is the spectrum of a sample that underwent all modifications without heme removal and DNPH reaction. The blue line is the same sample upon introduction of a heme-removing step. The black line is the sample treated for heme removal and reacted with DNPH.

Since the ELISA assay is particularly advised for heme-containing samples – as heme interferes with DNPH spectroscopic measurement – we introduced an additional step to allow measuring the carbonyl content on hemoglobin/myoglobin-rich kidney extracts by the Levine method (see Section 2). Without the heme removal step, the absorption contribution of heme groups in a non DNPH-treated sample dominates the 340–400 nm absorption range (Fig. 3, red line). The additional step for heme removal (see Section 2) fully eliminates the absorption contribution of the heme (Fig. 3, blue line), allowing for carbonylation to be easily detected when DNPH labelling is performed (Fig. 3, black line).

**Fig. 4:**
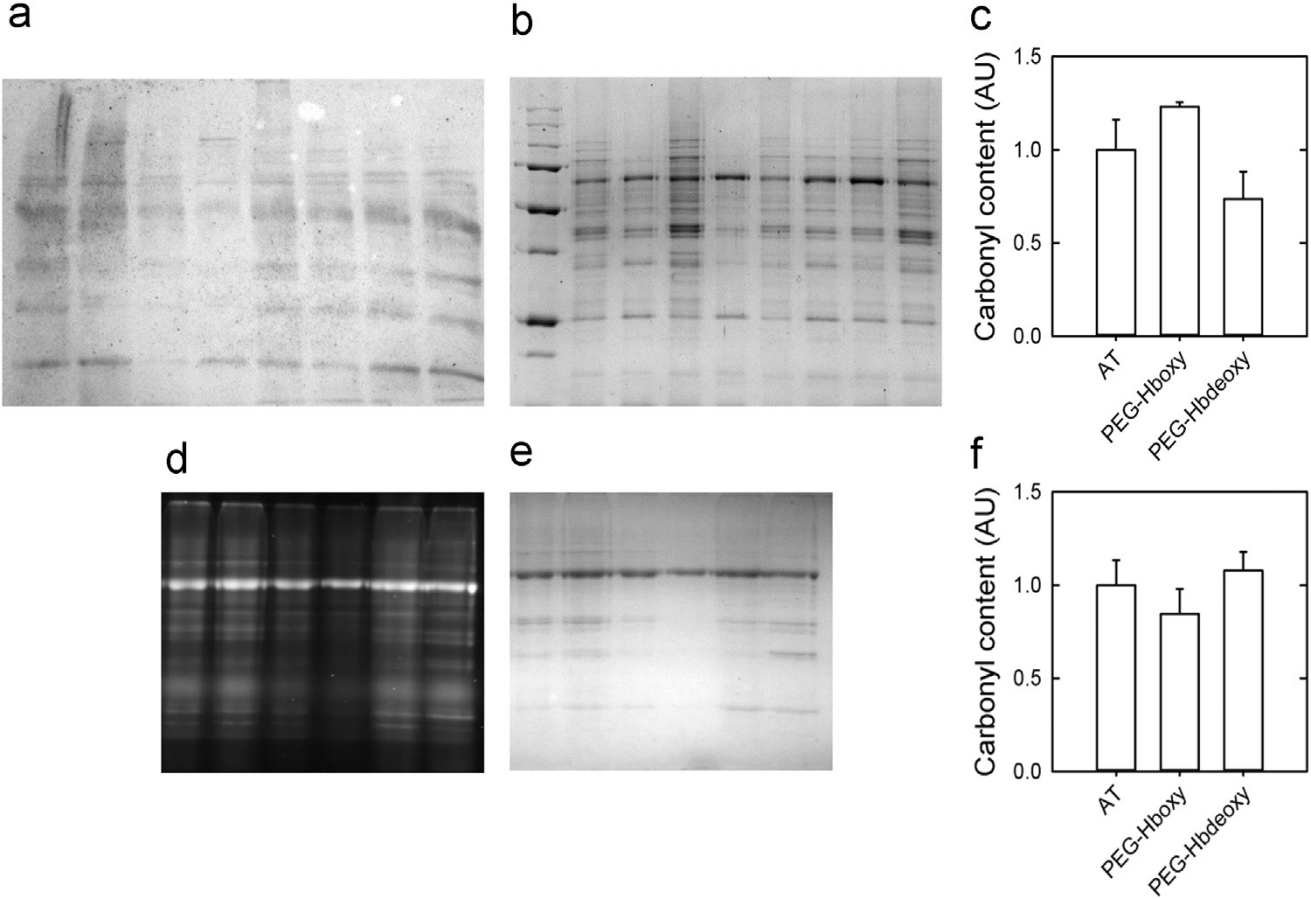
Results from Western blot (a–c) and in-gel FTC (d–f) detection of carbonyl content of heart extracts.

The results on the evaluation of carbonyl content with the two methods applicable after electrophoretic separation are shown in Fig. 4. Fig. 4a shows a Western blot developed with the Oxyblot assay and the corresponding SDS-PAGE used for data normalization (Fig. 4b). The total carbonyl contents estimated from the Western blot are reported in Fig. 4c. Fig. 4d–f represents the corresponding results with the fluorescein-5-thiosemicarbazide (FTC)-derivation method.

## 2 Experimental design, materials and methods

### 2.1 Sample preparation

Heart and kidney tissue samples were obtained from 14 Hartley Guinea pigs that underwent either autotransfusion or transfusion with PEG-Hb^oxy^ or PEG-Hb^deoxy^, two PEGylated hemoglobin-based oxygen carriers [1], [2], [3], [4], [5], [6] endowed with different oxygen affinity [2], as described in FRBM [11]. The aim of the work was to assess the degree of oxidative stress in the treatment groups in comparison with the control (autotransfused) group by determining the level of carbonylated proteins. No significant differences among groups were observed.

### 2.2 Detection of protein carbonylation

Samples for carbonyl detection were obtained from individual hearts and kidneys that were cryogenically ground with mortar and pestle and resuspended in a solution containing 50 mM phosphate buffer, pH 7.4, 1% DTT, in the presence of a protease inhibitor cocktail mixture (Sigma-Aldrich, St. Louis, MO, USA). The total protein content of the extracts, assessed using the Bradford assay (Bio-Rad Laboratories, Inc., Hercules, California, USA), was 1.2 ± 0.3 mg/mL.

#### 2.2.1 Levine spectrophotometric method

The carbonyl content of whole protein extracts was measured using the Levine method [7], [8], in which carbonylated proteins are derivatized with 2,4-dinitrophenylhydrazine (DNPH) to produce a chromophoric adduct exhibiting an extinction coefficient of 22,000 M^−1^ cm^−1^ at 366 nm. DNPH at 5 mM concentration was added to 500 μL aliquots of 1.2 mg/mL protein solutions and left to react for 1 h in the dark. Proteins were then precipitated with 10% trichloroacetic acid (TCA) and extensively washed with 10% TCA, followed by two washing steps with ethanol/ethyl acetate (1:1 v/v). The resulting pellet was solubilized in 6 M guanidinium hydrochloride. Spectra were collected in the 250–600 nm range, which allowed assessing both the protein concentration, from absorbance at 280 nm, and that of the DNPH derivatives, from absorbance at 366 nm. To take into account the contribution of heme proteins to the same absorption range as DNPH, the spectra of control protein samples that underwent all steps with the exception of DNPH derivatization were used as a reference. All spectra were normalized to the total protein content of each sample, as assessed by absorbance at 280 nm, assuming that one unit of absorbance corresponds to a protein concentration of 1 mg/mL. For kidney extracts, that exhibited a high heme content, a modified protocol was applied, which included an additional protein precipitation step with HCl-acetone (3:100 v/v) before addition of DNPH to remove the heme moiety [9].

#### 2.2.2 ELISA detection of carbonylated proteins

The OxiSelect™ Protein Carbonyl ELISA Kit (Cell Biolabs, San Diego, CA, USA) was applied to the same protein samples used for the Levine method in a 96-wells plate, according to the manufacturer׳s instructions. The chromogenic reaction catalyzed by horseradish peroxidase (HRP) was detected using a plate reader (Halo LED 96 – Dynamica Scientific Ltd., Newton Pagnell, UK). Calibration was performed using the calibrants provided by the manufacturer.

#### 2.2.3 Detection of protein carbonylation by western blot

For immunoblotting, samples containing 2 μg of protein were loaded onto two 12% SDS-PAGE gels. One gel was developed with Coomassie blue and the other was electroblotted to a nitrocellulose membrane with a Trans-Blot Turbo Blotting System (Bio-Rad Laboratories, Inc., Hercules, California, USA). The membranes were then incubated overnight at 4 °C in 2% BSA blocking solution and probed with a rabbit anti-DNPH antibody in a 1/150 dilution (OxyBlot™ Protein Oxidation Detection Kit, Merk Millipore, Darmstadt, Germany). The membrane was then probed with a goat anti-rabbit secondary antibody conjugated to HRP according to the manufacturer׳s instructions. Upon addition of the substrate CheLuminate-HRP PicoDetect (PanReac AppliChem, Darmstadt, Germany), the chemiluminescence signals were captured with a ChemiDoc^™^ system (Bio-Rad Laboratories, Inc., Hercules, California, USA). Western blot signals were normalized to Coomassie blue signals measured on the partner SDS-PAGE gel and expressed as arbitrary units.

#### 2.2.4 In-gel detection with fluorescein-5-thiosemicarbazide

In-gel detection upon derivatization with FTC was carried out using the protocol reported in [10]. Briefly, FTC was added in a ratio 1:4 v/v to protein solutions to give a 1 mM final concentration and incubated in the dark for 150 min at room temperature with gentle mixing every 30 min. Proteins were then precipitated with an equal volume of ice-cold 20% TCA (v/v). Mixtures were incubated at 4 °C for 10 min in the dark and centrifuged at 15,000 *g* for 10 min at 4 °C. Protein pellets were washed five times with ethanol/ethyl acetate (1:1, v/v) and resuspended overnight in the dark at 4 °C in 80 μL of a buffered solution containing 100 mM Tris–HCl, pH 8.0, 5 mM MgCl_2_, 1 mM EDTA, 8 M urea and 150 mM NaCl. Derivatized proteins (25 μg) were mixed with a loading buffer for SDS-PAGE and were separated on 15% SDS-PAGE gel. After electrophoresis, in-gel fluorescence of FTC-derivatized proteins was captured with a ChemiDoc^™^ system (Bio-Rad Laboratories, Inc., Hercules, California, USA) (*λ*_ex_ = 492 nm; *λ*_em_ = 516 nm). Total protein content was assessed on the same gel by Coomassie blue staining. FTC signals were normalized to Coomassie blue signals and expressed as arbitrary units.

### 2.3 Statistical analysis

Statistically significant changes were assessed by one-way analysis of variance (ANOVA) follow by Tukey׳s Test. In all analyses, a value *p* < 0.05 was taken as the level of statistical significance. In all plots, values are represented as mean ± standard error (SE). Graphs and statistical analyses were performed using the software SigmaPlot (Systat Software, San Jose, CA, USA).
