# Supplementary material for: Protein carbonylation detection methods: A comparison
Source: Data Brief. 2018 Jul 3;19:2215–20. doi: 10.1016/j.dib.2018.06.088 (PMC6141388; doi:10.1016/j.dib.2018.06.088)
Supplement: Supplementary file 1 — Supplementary material [file mmc1.docx]

Conflict of interest

All the authors confirm as No conflict of Interest.
